# Supplementary figures and images for: Local and population-level responses of Greater sage-grouse to oil and gas development and climatic variation in Wyoming
Source: PeerJ. 2018 Aug 14;6:e5417. doi: 10.7717/peerj.5417 (PMC6097500; doi:10.7717/peerj.5417)

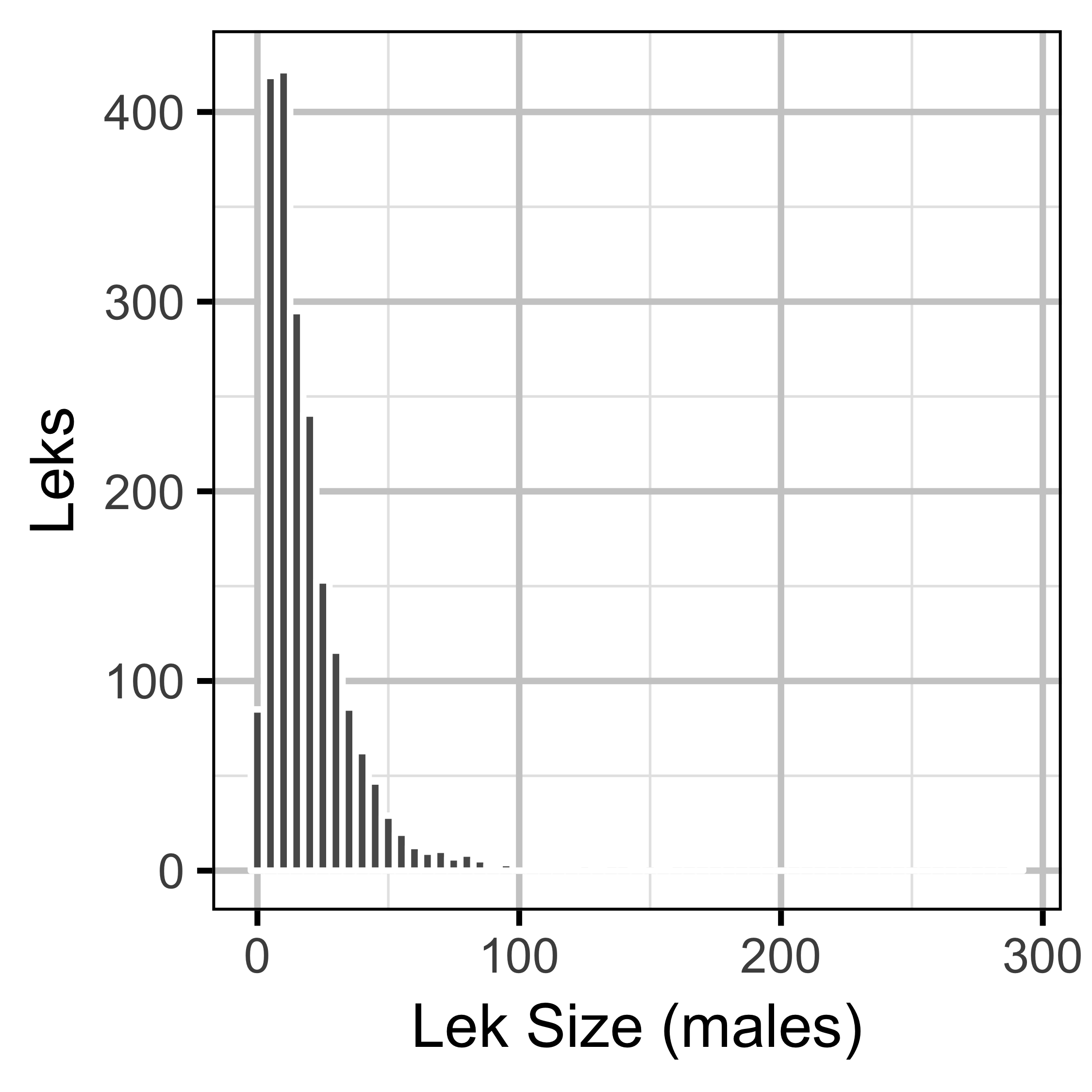

Supplement: Supplemental Information 1 [file peerj-06-5417-s001.png]

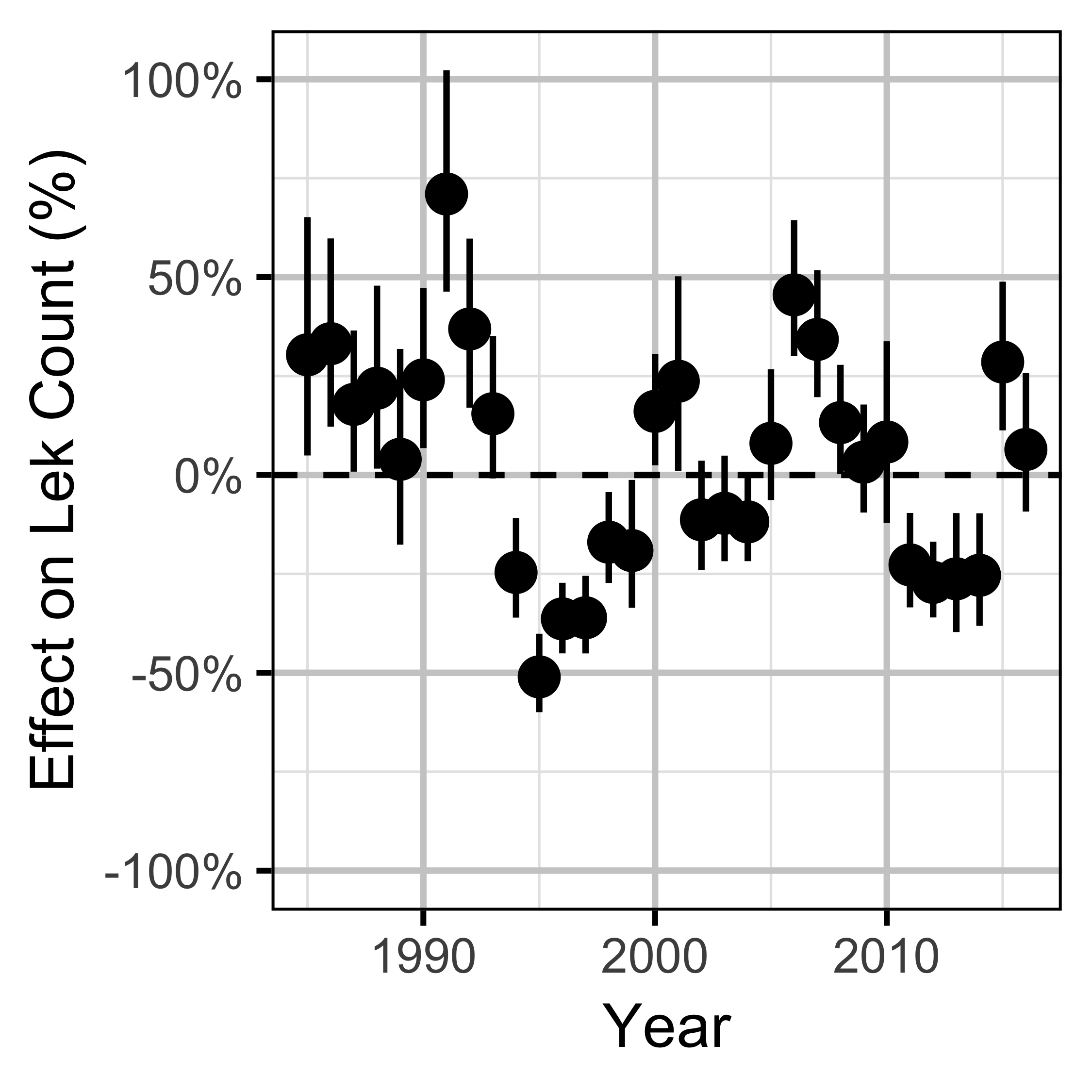

Supplement: Supplemental Information 2 [file peerj-06-5417-s002.png]

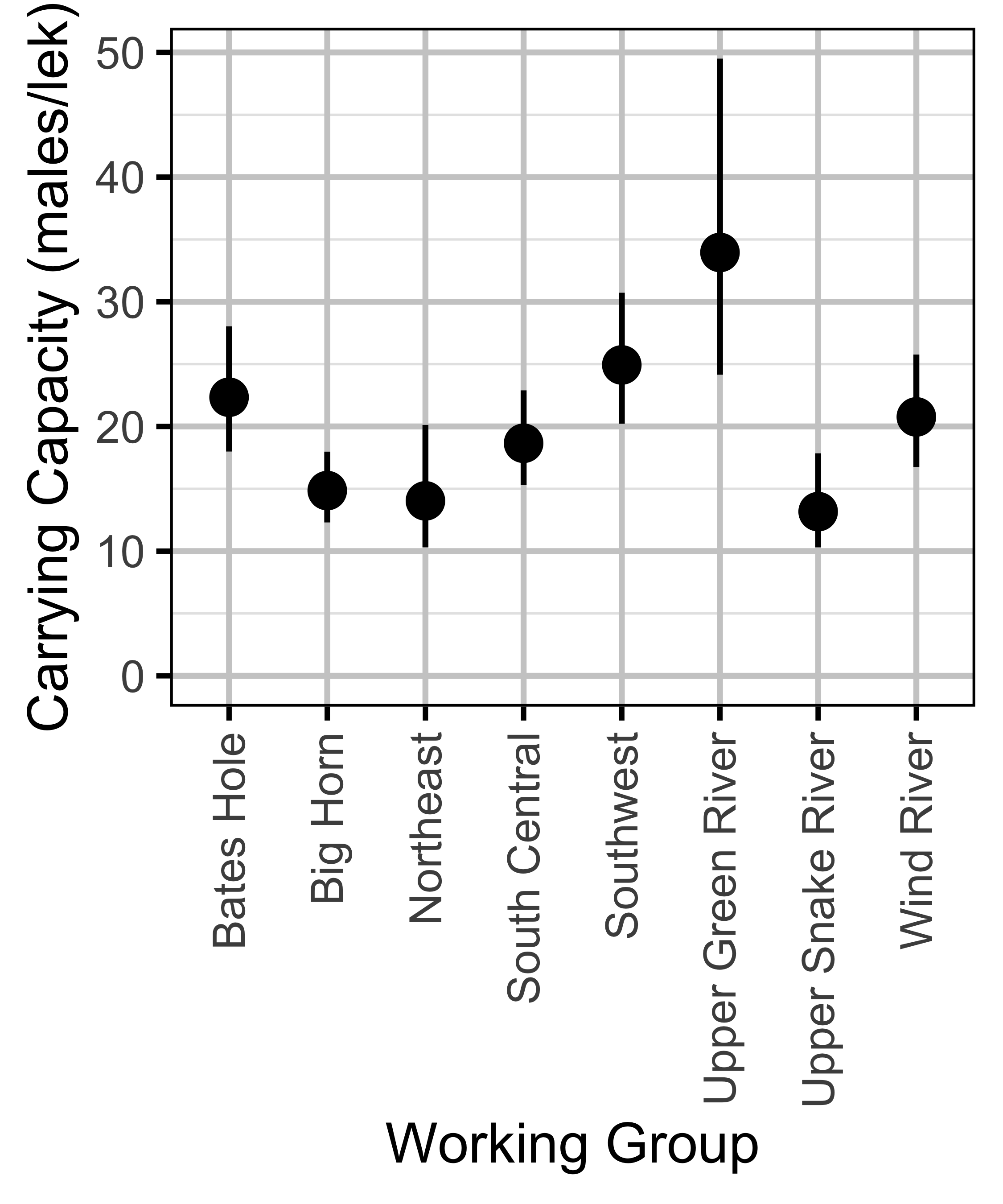

Supplement: Supplemental Information 3 [file peerj-06-5417-s003.png]

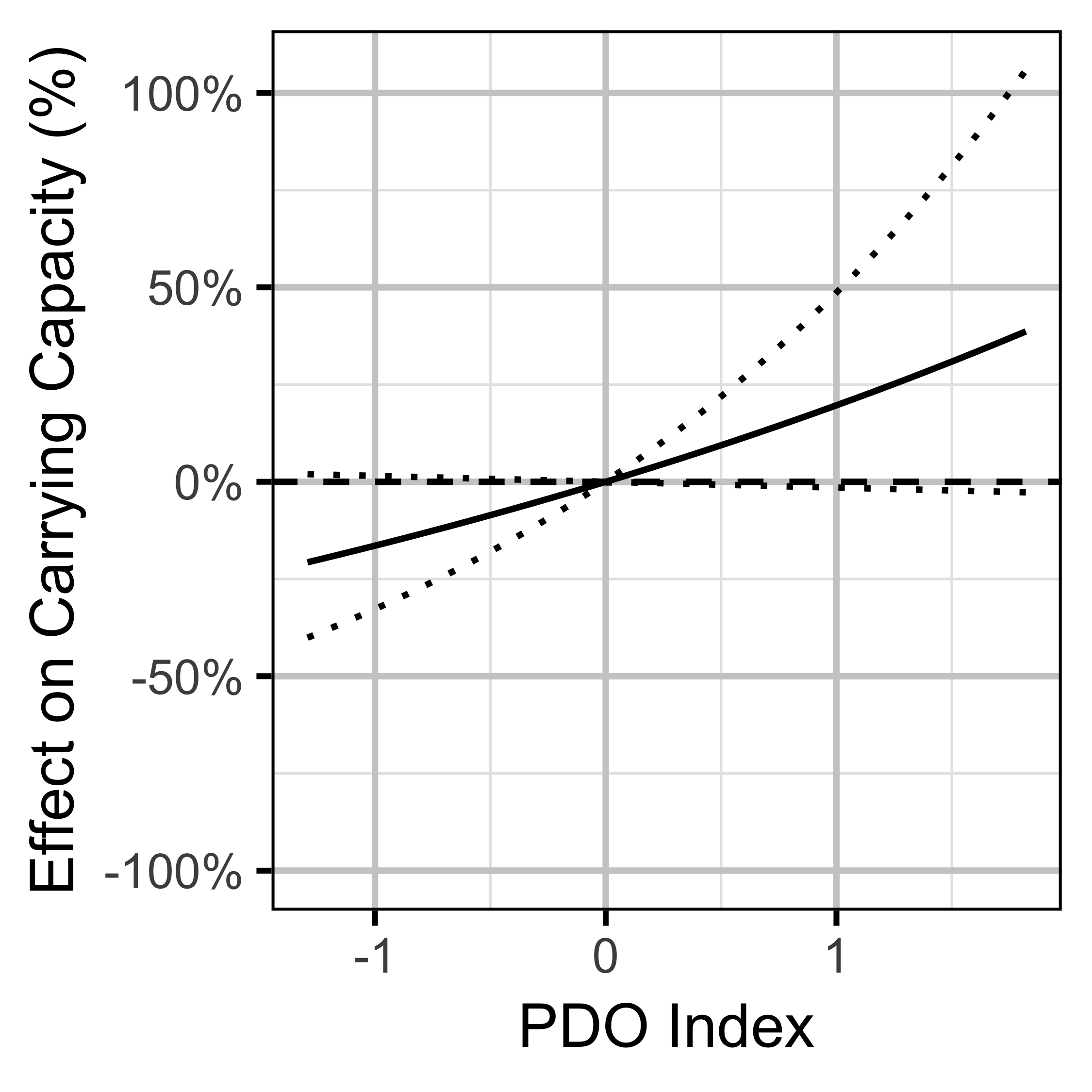

Supplement: Supplemental Information 4 — The effect is the percent change in the expected carrying capacity relative to a Pacific Decadal Oscillation index value of 0. [file peerj-06-5417-s004.png]

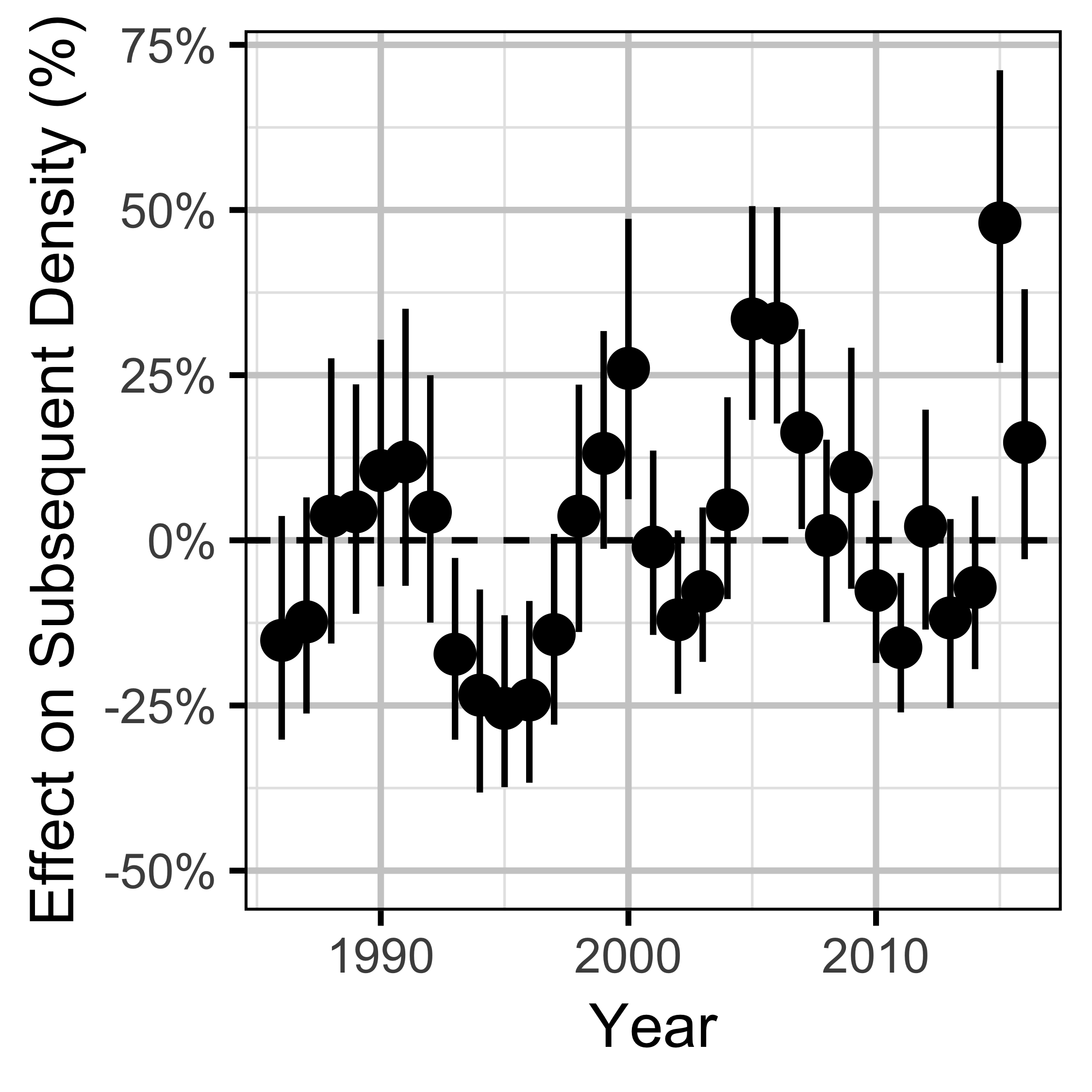

Supplement: Supplemental Information 5 [file peerj-06-5417-s005.png]

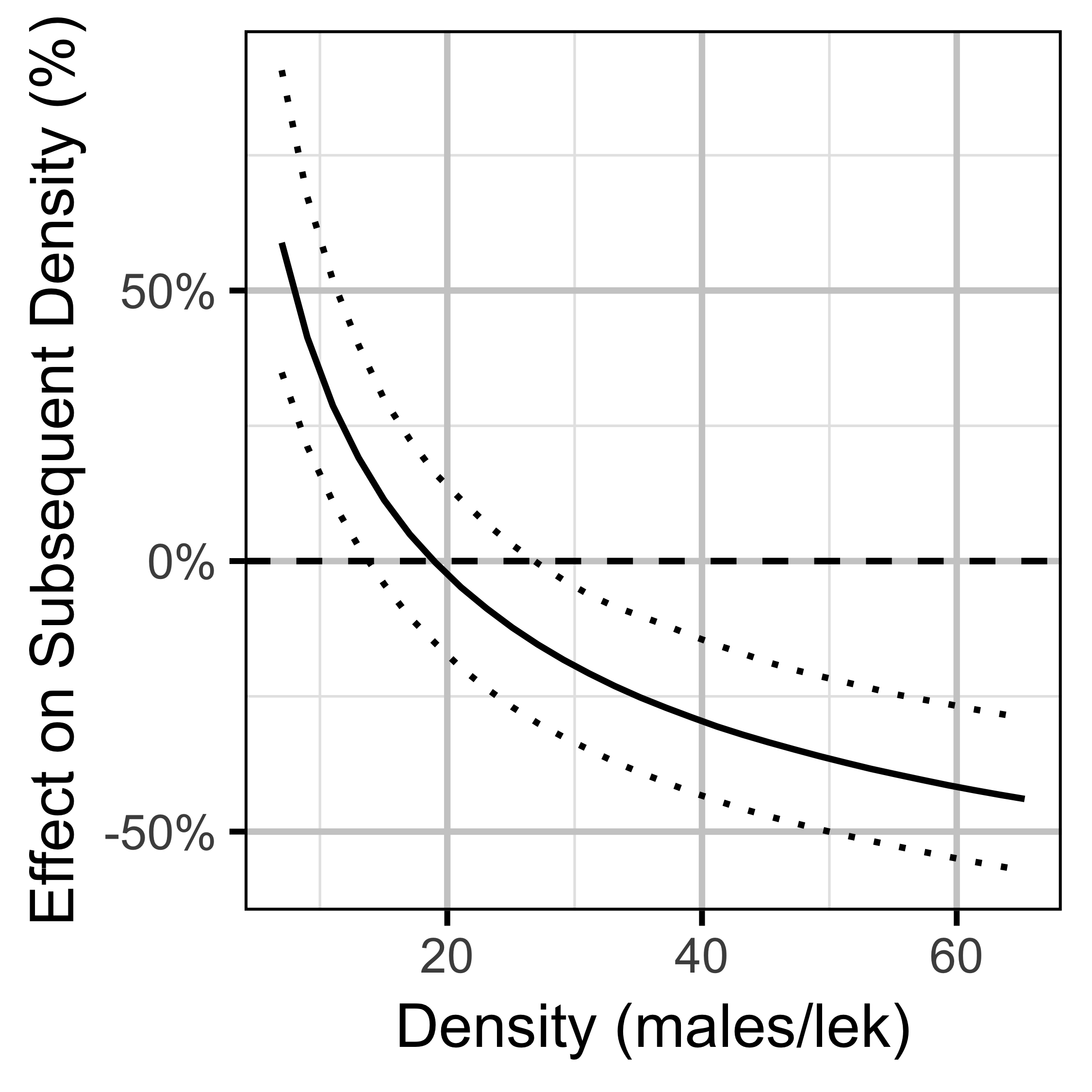

Supplement: Supplemental Information 6 [file peerj-06-5417-s006.png]
