# Supplementary material for: Local and population-level responses of Greater sage-grouse to oil and gas development and climatic variation in Wyoming
Source: PeerJ. 2018 Aug 14;6:e5417. doi: 10.7717/peerj.5417 (PMC6097500; doi:10.7717/peerj.5417)
Supplement: Supplemental Information 7 — The relative importance is across all models with the areal disturbance due to well pads and the Pacific Decadal Oscillation index both independently lagged one to four years. [file peerj-06-5417-s007.pdf]

Distance (km)	Models	Proportion	w_i
3.2	16	0.25	1.00
1.6	16	0.25	0.00
6.4	16	0.25	0.00
0.8	16	0.25	0.00

Table S1. The relative importance (w_i) of spatial scale as a predictor of the count of males sage-grouse at individual leks. The relative importance is across all models with the areal disturbance due to well pads and the Pacific Decadal Oscillation index both independently lagged one to four years.
