# Supplementary material for: Local and population-level responses of Greater sage-grouse to oil and gas development and climatic variation in Wyoming
Source: PeerJ. 2018 Aug 14;6:e5417. doi: 10.7717/peerj.5417 (PMC6097500; doi:10.7717/peerj.5417)
Supplement: Supplemental Information 8 — The relative importance is across all models with a lek distance of 0.8, 1.6, 3.2 and 6.4 km and the Pacific Decadal Oscillation index lagged one to four years. [file peerj-06-5417-s008.pdf]

Area Lag (yr)	Models	Proportion	w_i
1	16	0.25	0.99
2	16	0.25	0.01
3	16	0.25	0.00
4	16	0.25	0.00

Table S2. The relative importance (w_i) of the lag in areal disturbance due to well pads as a predictor of the count of males sage-grouse at individual leks. The relative importance is across all models with a lek distance of 0.8, 1.6, 3.2 and 6.4 km and the Pacific Decadal Oscillation index lagged one to four years.
