# Supplementary material for: Local and population-level responses of Greater sage-grouse to oil and gas development and climatic variation in Wyoming
Source: PeerJ. 2018 Aug 14;6:e5417. doi: 10.7717/peerj.5417 (PMC6097500; doi:10.7717/peerj.5417)
Supplement: Supplemental Information 9 — The estimates are for a lek distance of 3.2 km, areal disturbance due to well pads of one year and Pacific Decadal Oscillation index lag of two years. Model parameters are described in Table 1. [file peerj-06-5417-s009.pdf]

Parameter	Model	Estimate	SD	Lower	Upper
β_A	Average Maximum Likelihood	-0.210	0.015	-0.241	-0.180
β_A	Full Maximum Likelihood	-0.210	0.015	-0.241	-0.180
β_A	Full Bayesian	-0.210	0.015	-0.242	-0.182
β_P	Average Maximum Likelihood	0.164	0.054	0.059	0.270
β_P	Full Maximum Likelihood	0.168	0.049	0.072	0.263
β_P	Full Bayesian	0.166	0.052	0.067	0.264
β_0	Average Maximum Likelihood	2.491	0.058	2.378	2.605
β_0	Full Maximum Likelihood	2.490	0.057	2.378	2.603
β_0	Full Bayesian	2.488	0.062	2.370	2.615
$\log(\phi)$	Average Maximum Likelihood	-0.908	0.014	-0.935	-0.882
$\log(\phi)$	Full Maximum Likelihood	-0.908	0.014	-0.935	-0.882
$\log(\phi)$	Full Bayesian	-0.908	0.013	-0.934	-0.881
$\log(\sigma_L)$	Average Maximum Likelihood	-0.011	0.019	-0.048	0.026
$\log(\sigma_L)$	Full Maximum Likelihood	-0.011	0.019	-0.048	0.026
$\log(\sigma_L)$	Full Bayesian	-0.006	0.019	-0.044	0.030
$\log(\sigma_Y)$	Average Maximum Likelihood	-1.256	0.130	-1.512	-1.001
$\log(\sigma_Y)$	Full Maximum Likelihood	-1.260	0.128	-1.511	-1.008
$\log(\sigma_Y)$	Full Bayesian	-1.212	0.137	-1.448	-0.918

Table S3. The parameter estimates for the final lek count models with lower and upper 95% confidence/credible intervals. The estimates are for a lek distance of 3.2 km, areal disturbance due to well pads of one year and Pacific Decadal Oscillation index lag of two years. Model parameters are described in Table 1.
