# Supplementary material for: Local and population-level responses of Greater sage-grouse to oil and gas development and climatic variation in Wyoming
Source: PeerJ. 2018 Aug 14;6:e5417. doi: 10.7717/peerj.5417 (PMC6097500; doi:10.7717/peerj.5417)
Supplement: Supplemental Information 10 — The estimates are for a lek distance of 3.2 km, areal disturbance due to well pads of one year and Pacific Decadal Oscillation index lag of two years. Model parameters are described in Table 1. [file peerj-06-5417-s010.pdf]

Parameter	Model	Estimate	SD	Lower	Upper
β_A	Average Maximum Likelihood	-0.012	0.021	-0.052	0.029
β_A	Full Maximum Likelihood	-0.027	0.024	-0.073	0.020
β_A	Full Bayesian	-0.026	0.024	-0.072	0.020
β_D	Average Maximum Likelihood	-0.464	0.064	-0.589	-0.339
β_D	Full Maximum Likelihood	-0.464	0.064	-0.588	-0.339
β_D	Full Bayesian	-0.470	0.067	-0.604	-0.339
β_L	Average Maximum Likelihood	-0.304	0.043	-0.389	-0.219
β_L	Full Maximum Likelihood	-0.305	0.043	-0.389	-0.220
β_L	Full Bayesian	-0.306	0.044	-0.393	-0.222
β_P	Average Maximum Likelihood	0.056	0.044	-0.031	0.142
β_P	Full Maximum Likelihood	0.073	0.035	0.005	0.142
β_P	Full Bayesian	0.074	0.039	-0.007	0.146
β_0	Average Maximum Likelihood	1.351	0.179	1.000	1.702
β_0	Full Maximum Likelihood	1.352	0.178	1.002	1.702
β_0	Full Bayesian	1.370	0.185	1.019	1.731
$\log(\sigma_G)$	Average Maximum Likelihood	-3.006	0.310	-3.614	-2.398
$\log(\sigma_G)$	Full Maximum Likelihood	-3.030	0.314	-3.645	-2.414
$\log(\sigma_G)$	Full Bayesian	-2.906	0.354	-3.564	-2.172
$\log(\sigma_\eta)$	Average Maximum Likelihood	-0.489	0.175	-0.833	-0.146
$\log(\sigma_\eta)$	Full Maximum Likelihood	-0.487	0.175	-0.829	-0.145
$\log(\sigma_\eta)$	Full Bayesian	-0.474	0.178	-0.809	-0.117
$\log(\sigma_Y)$	Average Maximum Likelihood	-1.692	0.156	-1.997	-1.386
$\log(\sigma_Y)$	Full Maximum Likelihood	-1.708	0.152	-2.005	-1.410
$\log(\sigma_Y)$	Full Bayesian	-1.662	0.160	-1.969	-1.335

Table S4. The parameter estimates for the final population models with lower and upper 95% confidence/credible intervals. The estimates are for a lek distance of 3.2 km, areal disturbance due to well pads of one year and Pacific Decadal Oscillation index lag of one year. Model parameters are described in Table 1.
